# Laser-driven beam lines for delivering intensity modulated radiation therapy with particle beams

**DOI:** 10.1002/jbio.201200078

**Published:** 2012-08-29

**Authors:** Kerstin M Hofmann, Stefan Schell, Jan J Wilkens

**Affiliations:** Department of Radiation Oncology, Technische Universität MünchenKlinikum rechts der Isar, Ismaninger Str. 22, 81675 München, Germany

**Keywords:** radiotherapy, lasers, protons, particle beam therapy, laser ion acceleration, motion adaptation

## Abstract

**Abstract:**

Laser-accelerated particles are a promising option for radiation therapy of cancer by potentially combining a compact, cost-efficient treatment unit with the physical advantages of charged particle beams. To design such a treatment unit we consider different dose delivery schemes and analyze the necessary devices in the required particle beam line for each case. Furthermore, we point out that laser-driven treatment units may be ideal tools for motion adaptation during radiotherapy. Reasons for this are the potential of a flexible gantry and the time structure of the beam with high particle numbers in ultrashort bunches. One challenge that needs to be addressed is the secondary radiation produced in several beam line elements. (© 2012 WILEY-VCH Verlag GmbH & Co. KGaA, Weinheim)

## 1 Introduction

When treating cancer patients, radiotherapy is one of the common strategies besides chemotherapy and surgery. To improve the clinical outcome, the X-ray beam application methods developed from conformal techniques to intensity modulated radiation therapy 1 and to rotational therapy 2. Another promising option is to use charged particles (protons and heavier ions like carbon) for cancer therapy instead of photons due to their advantageous behavior when interacting with matter. The energy deposition of the particles increases with depth (up to a finite range) leading to a dose concentration in the tumor (at the Bragg peak) while minimizing damage of normal tissue 3, especially for advanced techniques like intensity modulated particle therapy. However, the current limitation in particle therapy is the high cost and the large space needed to build such facilities. This is due to the required large accelerators (synchrotrons or cyclotrons) and to the very complex beam lines needed to guide and deflect the particle beam to the patient, in particular rotating gantries that can direct the beam from any desired angle towards the patient. Therefore, various alternatives were investigated 4 with the aim of designing a more cost-efficient radiotherapy treatment unit like in photon therapy but with the physical advantages of particles. One of these methods is the acceleration of ions with lasers. Such a laser-based unit could, in the future, constitute a compact radiotherapy treatment system for cancer patients.

Laser ion acceleration works by shooting a high power laser onto a thin target, typically a foil of μm thickness 5. By hitting the foil the electrons of the target material are accelerated in a plasma and build up a high electric field due to charge separation. This field can yield up to 1 TeV/m and, therefore, extracts and accelerates the ions at the rear surface of the target. Hence, this regime is called target normal sheath acceleration (TNSA). With much thinner foils (nm) or higher laser intensities, other regimes can become more dominant as e.g. radiation pressure acceleration (RPA) or coherent acceleration of ions by lasers (CAIL) 5. At the moment, laser-driven protons reach energies up to about 60 MeV 6 which is not sufficient to treat cancer patients as these particles will stop very close to the skin. Therefore, many groups are investigating new accelerating mechanisms by varying the targets or the laser parameters with the goal of achieving particle energies up to 250 MeV (for protons).

Apart from the low energies, the laser ion acceleration systems operating so far show very different beam properties of the accelerated particles compared to conventional particle beams. One of the main differences is that the laser-driven beam is composed of ultra-short particle bunches (typically ns), which is in contrast to continuous or quasi-continuous pulsed beams from cyclotrons or synchrotrons. The pulse rate of the laser-driven particle beam is limited by the repetition rate of currently about 10 Hz of the laser and by the time needed to replace the target after each laser shot (if necessary). Furthermore, while conventional beams have a very narrow energy spread and consist of one particle type only, laser-driven beams have a broad energy spectrum showing an exponential decrease to higher energies and consist of a mixture of particles present within the target in the low energy region. However, compared to conventional machines, the laser-driven beams may offer a much higher number of particles per bunch permitting high doses for cancer treatments in short times.

These different beam properties give reason and opportunity to investigate new treatment techniques for radiotherapy. Therefore, the whole beam line has to be re-designed to use the laser-accelerated particles efficiently. This means not just copying the beam elements of the conventional systems but to utilize the beam more efficiently, with beam shaping elements and dose delivery mechanisms specifically tailored to the properties of laser-driven particles. The purpose of this paper is to present an overview over various potential delivery methods and the required beam elements for a laser-driven particle beam line. Moreover, we want to stress that laser-driven particle therapy in general not only offers a compact radiotherapy unit but also provides advantages for future adaptive radiotherapy treatments like gating or tumor tracking. These motion adapting techniques are under investigation in conventional particle therapy as well, since target movements are a big problem in external radiotherapy 7. Laser-accelerated particles promise effective gating therapies and easier to handle tracking methods.

## 2 Design of a treatment unit using laser-accelerated particles

**Figure 1:**
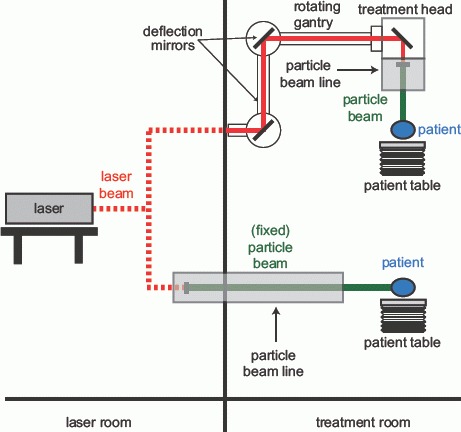
(online color at: www.biophotonics-journal.org) General setup of two scenarios of laser-driven particle therapy units. The upper beam line shows a compact gantry with mirrors guiding the laser beam to the treatment head containing a short particle beam line. The lower one is a fixed horizontal beam line which offers more space for the beam line elements.

To use laser-driven particles beams efficiently for radiotherapy one has to investigate new types of dose delivery techniques adapted to their properties (cf. 8). This includes techniques to deliver a homogeneous dose to the target volume in the patient while sparing the surrounding tissue and organs at risk as much as possible. Therefore, we gave consideration to a number of new conceivable dose application schemes and elaborated the required beam elements in the future beam line for each of these schemes. Such a beam line can be realized as a fixed beam or as a flexible one by using a rotating gantry directing the laser to the treatment head, which will then have to contain all necessary beam line elements in a very compact form. Both scenarios are illustrated in Figure 1. Since the laser beam is guided inside the gantry instead of the particle beam, the gantry can be designed very compact and heavy bending magnets as in conventional particle therapy are avoided. At the moment, the exact beam properties of laser-driven beams at high energies are not known yet. We will therefore present the beam delivery methods and beam line elements in a very general form and we will highlight the differences in the required beam line elements for each case.

## 2.1 Beam delivery methods

### 2.1.1 Static delivery

**Figure 2:**
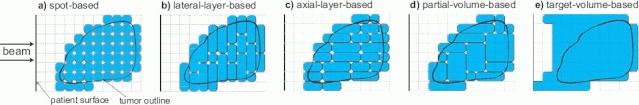
(online color at: www.biophotonics-journal.org) Sketch of five different dose delivery schemes, shown for one beam direction and a water equivalent patient. Each closed area is irradiated simultaneously within one laser shot.

Delivering a homogeneous dose to a stationary target volume can be achieved in various ways. In conventional particle therapy two delivery methods are used, namely “passive scattering” and “active scanning” 9,10. With laser-driven particles we have, in principle, more options than these two, illustrated in Figure 2. The different dose delivery methods are sketched for one beam direction and a water equivalent patient. For each method Figure 2 points out areas, so called clusters, in the tumor which are irradiated at once, i.e. with one single proton bunch 11.

Figure 2a demonstrates the spot-based delivery which is similar to the conventional active scanning technique. The target volume is irradiated with many individual spots by scanning the particle beam over the target volume. Each spot can be produced by one or more single laser shots of variable dose. This allows for the most flexible dose delivery with many degrees of freedom.

Figure 2b represents the lateral-layer-based delivery. This is a reasonable, efficient delivery if the fluence per bunch is very high or if the number of shots is limited due to a small repetition rate of the whole system. In case of the latter, we can save delivery time by covering a larger area in the target with one laser bunch, which in turn keeps the number of required shots low. In this method, the quasi-mono-energetic particle beam is spread laterally to cover a part of, or even a whole lateral layer in the tumor.

In contrast to this, one can also apply the dose in axial-layer-based clusters (Figure 2c). This concept arose from the fact that laser-accelerated particle beams occur with a broad energy spectrum anyway. Thus, the spectrum can be used without any modification 11 or can be modulated in any user-defined way. For instance, it may be possible to deliver a whole spread-out Bragg-peak (SOBP) within one laser shot 12. Here the narrow beam with the broad energy spectrum is scanned over the target and delivers a certain dose in a cluster of arbitrary axial length.

Another conceivable delivery method would be a combination of both clustering methods, namely the partial-volume-based delivery (Figure 2d). This method combines the two layer-based clustering schemes, i.e. particle beams with broad energy spectra are, in addition, spread laterally. This combination leads to bigger clusters in the target volume and, therefore, to even faster deliveries. Furthermore, more particles can be utilized for therapy in case of high particle numbers per shot, which improves the overall efficiency of the beam line.

Going from partial volumes to the whole target volume, it is also possible to irradiate the whole tumor within each laser shot. For such a delivery, the beam must be broadened and collimated afterwards. And, independent of whether the spectrum of the beam is broad or narrow, one must conform the range of the particles to the distal edge of the tumor. This technique (Figure 2e) is very similar to the conventional passive scattering technique.

In summary, the delivery methods presented in Figure 2a and e correspond to conventional delivery technique, whereas Figures 2b–d represent alternative delivery methods especially customized for laser-driven particles. All of these delivery methods except the target-volume-based method (Figure 2e) allow a superposition of partial volumes or partial layers with different numbers of particles. Therewith, one can achieve an intensity or fluence modulation. This means these laser-driven dose delivery methods offer the opportunity to apply intensity modulated particle therapy 13.

### 2.1.2 Motion adaptation

The presented dose delivery methods assume static targets. However, the target volume can move or drift during therapy, for example due to the breathing of the patient. Hence, also for laser-driven therapy one must consider adaptation of the treatment depending on the target motion. In photon therapy approaches like gating 14 or tumor tracking 15 are under investigation. For particle therapy besides gating and tracking, re-scanning or re-painting represent an additional option in clinical practice 16–18. These techniques are conceivable for laser-accelerated particles, too. Moreover, with laser-driven particles one could realize motion adaptive radiotherapy more easily and more efficiently. The reason is the potentially high fluence per bunch which is beneficial for all adaptive techniques. Another advantage could be the separation of motions which becomes possible with the flexible laser gantry and additional scanning magnets. Then, one could separate the scanning motion, i.e. the movement between two spots or two clusters, and the tumor tracking motion, i.e. following the target with the beam, into two independent systems, which do not pose any constraint on each other.

## 2.2 Beam line elements

To build a laser-accelerated particle (LAP) beam line for radiotherapy various beam line elements have to be utilized. These can be grouped into three categories, namely beam transport, beam shaping and patient safety elements. This chapter presents the required devices depending on the used delivery method.

## 2.2.1 Beam transport

**Figure 3:**
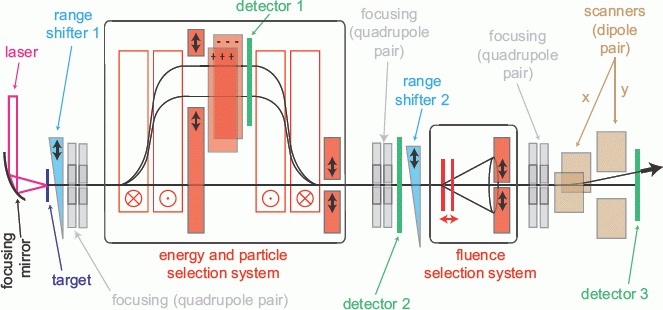
(online color at: www.biophotonics-journal.org) Simple assembly of beam line elements that are required in a laser-driven particle therapy unit.

**Figure 4:**
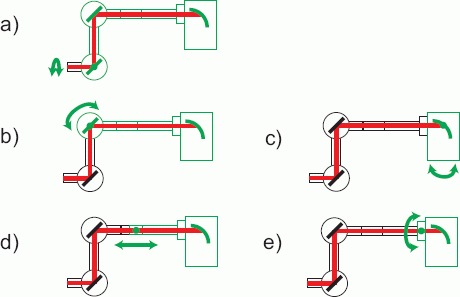
(online color at: www.biophotonics-journal.org) Possible rotations and elongations of the gantry leading to many degrees of freedom for patient treatment (“gantry scanning”). Black parts do not move, while green parts can move. Subfigure (a) shows a normal gantry movement whereas (b) and (c) demonstrate a tilt at different parts of the gantry. In (d) an elongation is illustrated and (e) represents a rotation of the treatment head around the beam axis.

Once particles are accelerated by the laser, the beam produced must be guided and shaped on its way to the patient. To avoid a diverging beam which would be undesirable in the spot-based and the axial-layer-based delivery, the beam could be focused with quadrupoles, for instance, like illustrated in Figure 3 (in particular if the beam is nearly monoenergetic). Scanning magnets can be used to deflect the beam to different spots or clusters, if necessary. In a LAP gantry, however, this scanning can also be accomplished by simple movements of the treatment head, which we call “gantry scanning”. This flexibility is possible since the laser is directed with mirrors inside the gantry, permitting an easy elongation or rotation by just adjusting the mirrors, rather than having to adjust bending magnets for the particle beam. Possible variations are shown in Figure 4a, where a shows the normal gantry movement by a rotation of the whole system and Figure 4b–e illustrate additional movements possible with a LAP gantry. More precisely, Figure 4b and c represent a feasible tilt of either the front gantry arm or the treatment head by a tilt of the respective mirror and Figure 4d shows the elongation (or shortening) of the gantry arm. Besides the rotation of the whole gantry a rotation of the treatment head around the laser beam axis is also possible as indicated in Figure 4e. Even more flexibility is achieved when using the patient table as a further degree of freedom. Whether this “gantry scanning” is feasible in a real patient treatment unit and how fast its performance will be depends strongly on the required beam line elements.

Another, more complex device that could be necessary is a focusing and beam transport system for a cone shaped, divergent particle beam with a broad energy spectrum. This is a demanding task, especially with the condition to be efficient in collecting a high particle number. One solution for this could be the use of pulsed solenoids to capture the particles 19.

## 2.2.2 Beam shaping

To shape the beam individually for each patient, some beam elements equal to those of a conventional particle therapy beam line can be used like scatter foils, energy degraders/range shifters (cf. Figure 3) or collimators. However, especially in a LAP system one could consider an adaptable collimator instead of a fixed one. This could either be a multi-leaf collimator like those clinically utilized for intensity modulated photon therapy or a circular collimator with an adjustable diameter (see e.g. Ref. 20).

Besides these conventional components, we also need specific ones to perform patient treatment with laser-accelerated particles. Most of them were already described elsewhere 9,12,13, and they are just presented shortly here:

Energy Selection System (ESS): As the energy spectrum of the particle beam can be broad, an ESS could be required. Such a system as proposed by Fourkal et al. 21,22 can be seen in Figure 3. One possible setup of an ESS consists of four magnetic dipole fields and two variable beam blocker pairs. The magnetic fields force the particles to separate in the central plane depending on their energy. Then, the beam blockers can cut certain particles with selectable energies due to this separation in energy. Depending on the settings a mono-energetic beam or a (small) part of the incoming spectrum exits the ESS at the second beam blocker. Alternatively, a quadrupole doublet or a solenoid followed by a small aperture may also serve as an ESS.

**Energy Modulation System (EMS):** In order to modulate the energy spectrum, one can additionally add scattering material in the central part of the ESS like a wedge 12. Then, particles with different energies transit different thicknesses of scattering material. Particles which are deflected too far from the optimal trajectory cannot exit through the second beam blocker. This allows controlling the number of particles per energy bin. Conventional modulator wheels cannot be utilized within a single particle bunch (with a bunch duration of typically 1 ns), but could be employed in a scenario with a certain number of shots for each step of the modulator wheel.

**Particle Selection System (PSS):** An ESS can be expanded to a PSS by including electric fields to select particles with a desired charge per energy ratio. If a mono-energetic beam will be selected by the ESS, one electrode constitutes the simplest way to realize a PSS (see Figure 3). Since the particles are deflected in the electric field, the downstream system has to be bent slightly to ensure that the required particles are guided to the exit of the ESS. This system can, for example, be adjusted to just supply protons if the laser-driven beam consists of a mixture of particles. If the transport of a broad energy spectrum is required, the PSS gets more complicated.

Fluence Selection System (FSS): Depending on the number of particles per shot, a further element could be needed to regulate the fluence. This could be useful in case of very high particle numbers per shot or if a spatial shape of the beam is explicitly wanted. Even if several shots are required for one cluster, a FSS could be reasonable. A realistic example would be a case where the needed fluence is not an integer multiple of the available fluence per shot. In such a case, the last shot needs to be down regulated in its particle number. The implementation of a FSS, therefore, could spread the beam using a scattering foil or by modifying the focusing magnets. Then, one could cut the number of particles with a simple or a multileaf collimator, as shown in Figure 3.

## 2.2.3 Patient safety

Since the laser-driven particle accelerator is used for patient therapy, we definitively need safety elements like detectors to monitor the beam and shielding to avoid exposure of the patient by secondary radiation. Such detectors (cf. Figure 3) would monitor the total fluence, the beam position or the energy spectrum to ensure the correct treatment. To find appropriate detectors which can measure high peak fluence rates in real-time is a remaining challenge.

## 2.2.4 Possible configurations of a laser-driven particle beam line

Depending on the beam properties and the chosen delivery method, certain beam elements of the ones explained above must be included in the LAP-beam line. This is demonstrated in Table 1. The elements listed in “independent of beam properties” of Table 1 are required by the respective delivery method. The beam line elements dependent on the beam properties are displayed in the lower part of Table 1. Here, we considered the particle types, the fluence and the energy, but we only present those cases in which additional devices have to be added. For instance, when the beam consists of several particle types, a PSS could choose just protons for patient treatment. In case of high fluences per shot, the spot-based and the axial-layer-based delivery would also require an FSS in the beam line. The energy properties we considered are either broad or narrow energy spectra and with the maximal energy being fixed or variable. It is clear that in case of broad energy spectra the ESS is needed, independent whether the maximal energy is fixed or variable. For fixed and narrow energy spectra we definitely need an energy degrader (DEG) to adjust the range in the patient. When the energy is fixed but with a broad spectrum, the ESS takes the function of the degrader and, therefore, no additional degrader is needed. Depending on the delivery an EMS is either required or optional.

**Table 1:** Required beam elements for the LAP-beam line depending on the dose delivery schemes (columns). For each delivery method the required elements are given dependent or independent of the beam properties. This table only shows beam elements dependent on either the delivery or the beam properties. Mandatory devices (e.g. for patient safety) which are needed in any case are not listed here.

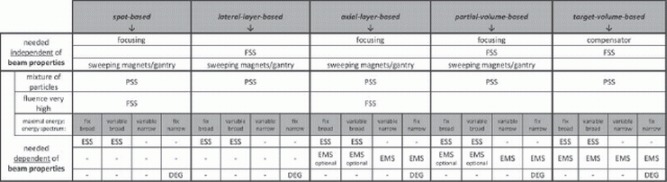

Table 1 demonstrates also the range of possible variations in the beam line. Going from left to right (i.e. from spot-based delivery to target-volume-based delivery), more and more elements are required in the beam line. Another important factor is the initial energy spectrum, which strongly influences the required beam line. This stresses that the final beam line setup is highly dependent both on the beam properties and the desired dose delivery method.

## 2.2.5 Supplements for adaptive radiotherapy

Table 1 does not include adaptive therapy deliveries. If motion management is an issue, a very important treatment room component is an imaging system to observe the target motion. Beside this, maybe sweeping magnets and additionally a flexible gantry system to allow for “gantry scanning” (see Figure 4) is reasonable. Furthermore, a fast energy adaptation is needed. This could be a normal degrader but it must be fast enough to adapt the energy to the desired depth in the patient in real-time, as this would be an issue especially in tracking (cf. 23).

## 3 Promises and challenges

In summary, laser-accelerated particles do not only offer more possibilities to deliver dose to cancer patients, but they also provide potential advantages. Due to potentially high particle numbers per bunch and the possibility to irradiate larger areas in the tumor simultaneously (up to a full spread-out Bragg peak in a single shot), the delivery may be very fast. Another advantage can be seen in motion adaptation. In a gating scenario, the very short bunch length and the high fluence per bunch offers the possibility to deliver a high dose in one single gating period. This could shorten the delivery time enormously because only few or even only one gating period is needed to deliver the whole desired dose.

For tumor tracking these time and fluence advantages also take place. One could even argue that almost no tracking is needed when the whole dose is delivered in a time in which the tumor nearly does not move. Nevertheless, we also see an advantage during the active tracking stage. Having the flexible and easily adaptable LAP-gantry one can separate the scanning motion from the tracking motion. This would at least simplify the control software of the motion adaptation.

When considering the delivery methods, each one offers its own advantages and disadvantages. In some patients the spot-based delivery would be beneficial as it offers the highest degree of freedom during treatment plan optimization. This is profitable for tumors situated close to sensitive organs at risk, even if delivery times become longer. In other cases, e.g. for small target volumes far away from organs at risk, the target-volume based delivery could show advantages in spite of the compromised dosimetric quality. For certain cases, this delivery method might be fast enough that target motion does not play a role. Thus, these examples stress that the best delivery method for the patient cannot be found generally, but should be chosen individually depending on the location of the tumor. In addition, the choice of delivery methods also depends on the final beam properties. Although one could implement almost every delivery method for each set of beam parameters, not all combinations are reasonable, especially with respect to secondary radiation produced in the beam line.

In any case it would be desirable to have a simple beam line with not too much and not too complicated elements. For instance, it would be ideal to exclude the ESS because of the secondary radiation produced there. An ESS compromises the efficiency of the whole system and requires heavy shielding, especially for neutrons. Hence, the optimal particle beam to be generated by the laser would be a nearly mono-energetic beam which would allow one to skip the whole ESS. Another advantage of a treatment head without an ESS is the more realistic possibility to perform the proposed “gantry scanning”. Then, the LAP treatment unit with a fast gantry scanning can be considered the particle beam analogy to dynamic X-ray treatment units like the gimbaled Vero system 24 or the robotic CyberKnife 25.

Independent of the beam line elements, but strongly dependent on the number of particles per laser shot, the repetition rate of the whole treatment system could also constitute a challenging parameter. In particle therapy the required dose in the target volume corresponds to a certain particle number to be deposited in this volume. Hence, the treatment time (which should not exceed 15 minutes) is given by the number of shots required to deliver the required number of particles and the repetition rate of the system. As the number of shots depends on the chosen delivery method and on the (currently not known) number of particles in one shot, it is uncertain whether a repetition rate of 10 Hz is sufficient for patient treatment. In a former work 11 we optimized proton treatments in a treatment planning software to investigate the efficiency of the clustering methods for a head and neck cancer case (target volume: 285 cm^3^). For a supposed number of about 10^8^ protons per shot, we found shot numbers in the order of a few thousand for clustered deliveries (lateral-layer-based or axial-layer-based) and up to almost 70000 for spot-based deliveries without explicit optimization of the number of shots. These results point out the efficiency of the clustered deliveries and show that a repetition rate of 10 Hz might be enough for patient treatments with a fluence of 10^8^ particles per shot. If this number is lower, higher repetition rates are required.

## 4. Conclusion

In general, laser-driven particles are a promising option for future radiation therapy. This is not only due to the potential of compact and cost-efficient treatment units, but, in addition, they may provide new delivery methods, in particular with respect to motion adaptive radiotherapy. Over the next years, the optimal design of laser-driven particle beam lines has to be further developed along with the experimental progress on the laser ion acceleration schemes and rising particle energies. As soon as the properties of a high energy, laser-accelerated beam are known, one would pick a design and simulate the particle transport more thoroughly, as currently done for low energies 26. At the moment such a simulation at high energies would be too speculative as the input parameters are not known. In the meantime, radiobiological experiments 27–30 will continue to complement these developments to evaluate the potential of laser-driven particle beams with respect to their biological effects. Overall, this concept is an interesting application of lasers in medicine which needs further investigation but may offer a unique tool for cancer therapy.

Kerstin Hofmann studied physics at the University of Heidelberg from 2005–2011 and did her diploma thesis at the German Cancer Research Center in Heidelberg in the field of medical physics. Since October 2011 she works on her Ph.D. thesis at the Klinikum rechts der Isar of the Technische Universität München as a student of the International Max Planck Research School of Advanced Photon Science (IMPRS-APS).

Stefan Schell studied physics at the University of Heidelberg and the University of Oregon in Eugene (OR, USA). He wrote his diploma thesis at the German Cancer Research Center in Heidelberg about radiobiological models for radiation treatment planning, and his Ph.D. thesis at the Technische Universität München about treatment planning for laser-driven particles. Since 2011 he works for a company manufacturing treatment planning solutions.

Jan J. Wilkens studied physics in Munich and Nottingham (GB). He received his Ph.D. from the University of Heidelberg and was a postdoctoral researcher at the German Cancer Research Center in Heidelberg and at Washington University in St. Louis, USA. In 2008, he became professor for “Advanced Technologies in Radiation Therapy” at the Technische Universität München. His research interests focus on optimization methods in treatment planning and new technologies for high-precision radiotherapy.
